# *In-vitro* Evaluation of Antioxidant and Antibacterial Potential of GreenSynthesized Silver Nanoparticles Using *Prosopis farcta* Fruit Extract

**Published:** 2019

**Authors:** Sepideh Salari, Sedigheh Esmaeilzadeh Bahabadi, Alireza Samzadeh-Kermani, Forough Yosefzaei

**Affiliations:** a *Department of Biology,* *Faculty of Basic Sciences, University of Zabol, Zabol, Iran. *; b *Department of Chemistry,* *Faculty of Basic Sciences, University of Zabol, Zabol, Iran. *; c *Department of Biology, Faculty of Science Urmia University, Urmia, Iran.*

**Keywords:** Antioxidant activity, Antibacterial properties, Prosopis farcta, Silver nanoparticles

## Abstract

Nowadays, green synthesis of metal nanoparticles has become a promising synthetic strategy in nanotechnology and materials sciences. In this research, biosynthesis of silver nanoparticles (AgNPs) was successfully accomplished in the presence of *Prosopis farcta* fruit extract as a reducing agent. Proceeding of the reaction was assessed by using UV–vis spectroscopy. Characterization of silver nanoparticles was carried out by X-ray Diffraction spectroscopy (XRD) and transmission electron microscopy (TEM). The influence of process variables such as temperature, reaction time, and extract concentration was also investigated to optimize the biosynthesis of silver nanoparticles. The average size of synthesized AgNPs was 12.68 nm (10.26-14.65 nm). Furthermore, fruit extract and AgNPs were evaluated for total phenolic and flavonoid contents and were subjected to determine their antiradical scavenging activity using 1,1-diphenyl-2-picryl-hydrazyl (DPPH) and ferric reducing antioxidant power (FRAP) assay and antimicrobial activity against *Staphylococcus aureus*, *Streptococcus pneumonia*, *Escherichia*, *Salmonella typhi* using the disk diffusion method. The total phenols and flavonoids in AgNPs-containing plant extract were 462.69 (mg GAE/g extract) and 386.94 (mg QE/g extract) respectively, which were significantly higher than fruit extract. Biosynthesized AgNPs showed a higher antioxidant and antibacterial activity compared to* P. farcta* fruit extract alone. It could be concluded that *P. farcta* fruit extract can be extensively used in the production of potential antioxidant and antibacterial AgNPs for biomedical application.

## Introduction

Nanotechnology has been emerged as one of the most active fields of research in material science over the last decades. The fact that nanoparticles (NPs) exhibit a number of interesting and unique properties owing to their specific characteristics, such as size, distribution and morphology, leads themselves to a wide variety of applications (1). Among noble metal nanoparticles, silver nanoparticles (AgNPs) have a wide area of interest since they have too many applications in difference fields such as in the fields of dentistry, clothing, catalysis, mirrors, optics, photography, electronics, and the food industry. Because of these applications, many methods for synthesis of AgNPs have been developed. These methods must have capable to control the size of AgNPs. Therefore, the AgNPs with small particle size and without bulking between particles is favorable (2). Recently, several chemical and physical routes and green synthesis are known for preparation of metal nanoparticles (3). However, many of these routine methods are very costly and toxic to the environment (4). On the other hand, the biological method was reported as clean, non-toxic and environmentally acceptable route (5, 6). In this regard, the use of plants and its extracts as reducing agents for the production assembly of AgNPs is very feasible, cost-effective, and eco-friendly (7). Biomolecules like protein, phenols, and flavonoids in plants not only play a role in reducing the ions to nano size, but also play an important role in capping of nanoparticles (8). Fruit extracts from various plants have been used to produce AgNPs, such as *Nothapodytes nimmoniana* (9),* Crataegus douglasii *(10), *Garcinia mangostana *(11),* Averrhoa carambola *(12)*, Lantana camara* (13)*, Emblica officinalis* (14). *Prosopis farcta* (Leguminosea family) is a native plant of United States, northern Africa, and Asia (15). It grows in southern Iran in the Sistan and Baluchestan, Hormozgan, Bushehr, Khuzestan and southern Fars provinces (16). *P. faracta* is a good source of phenolic compounds and flavonoids such as rutin, myricetin and caffeic acid derivatives (17)*. P. farcta* have been used as a traditional medicine for the treatment of some diseases and disorders. Antioxidant capacity (18), antimicrobial (19), and antitumour activity of this plant (20) have been reported recently. Hence, in the present study we have demonstrated biosynthesis of AgNPs by reduction of aqueous silver nitrate (AgNO3) using *P. faracta* aqueous fruit extract for their potential antioxidant and antibacterial effects. 

## Experimental

*Chemicals*

Silver nitrate (99.98%), ammonium hydroxide (25%), hydrochloric acid (37%) and nitric acid (70%) were purchased from Merck Company (Darmstadt, Germany). 1,1-diphenylpicrylhydrazyl (DPPH) was purchased from Sigma Aldrich. All chemical materials were of analytical grade and were used without any further purification. All solutions were freshly prepared using double distilled water and were kept in darkness to avoid any photochemical reactions. All glass ware which were used in the process, were washed with acetone, and then with double distilled water and dried before use.

*Plant extract*

*Biosynthesis of silver nanoparticles*

*P. farcta* fruits were collected in June 2015, from Zabol, Sistan, and Baluchestan province, Iran. The fresh fruits were thoroughly washed with deionized water for several times and then dried. 2.5 g of fruit was added to 100 mL of distilled water and boiled in for 30 min. The prepared suspension was filtered by Whatmann No. 40 filter paper. The resultant solution was centrifuged at 4000 rpm for 30 min in order to remove impurities. 9 mL of 0.001 M aqueous solution of silver nitrate (AgNO_3_) was prepared in a stoppard erlenmeyer flask and then different volumes (100, 110, 120, 130, or 140 µL) of fruit extract (2.5 g/100 mL) was added to the flask at room temperature in darkness. To study the influence of temperature on nanoparticle synthesis, the reaction mixtures were incubated in a rotary shaker at 150 rpm at 50, 60 or 70 °C for a period of time. After the reaction was completed, the mixture was centrifuged for 20 min at 15000 rpm. The precipitate was washed with distilled water and centrifuged several times in order to remove impurities like starting material or natural products which still remained from extract. The influence of reaction time was evaluated by incubating the reaction mixtures with optimum composition for 25 or 45 min.

*Optimization study*

In order to optimize the synthesis of AgNPs, the influence of every variable on the reaction process including reaction temperature, extract concentration (volume) variation, and the reaction time were evaluated separately, as one variable held constant and the others varied.

*Influence of extract concentration*

Different volumes of aqueous fruit extract of *P. farcta* (100, 110, 120, 130 and 140 µL) were selected after preparing the extract by boiling freshly cut fruits in deionized water for 2 min. Then, AgNO_3_ (0.001 M) solution was added and incubated for 4 h in rotatory shaker at 150 rpm.

*Influence of reaction temperature*

In order to find the influence of reaction temperature on the biosynthesis of silver nanoparticles, different concentrations of aqueous fruit extract of *P. farcta* at different reaction times were investigated for three temperatures of 50, 60, and 70 °C separately.

*Characterization*

*UV-vis analysis*

The absorption spectrum of the reaction mixture was recorded at room temperature by using UV–vis spectrophotometer (Rayleigh, UV-2100) at the resolution of 1 nm.

*XRD analysis*

The structure of silver nanoparticles was determined by X-ray diffraction using the Bruker – D8 Advanced (Germany) instrument. A wavelength (λ) 0.15418 nm was used for these analyses. The XRD patterns were recorded at the scan speed of 2° min^−1^.

**Table 1:** Total phenolics and flavonoid contents of synthesized AgNPs and fruit extract of *P. farcta*

**Samples**	**Total phenolics (mg GAE/g extract)**	**Flavonoids (mg QE/g extract)**
AgNPs	462.69 ± 3.42	386.94 ± 3.24
Fruit extract	366.21 ± 3.03	283.33 ± 3.09

**Table 2:** DPPH radical scavenging activity of *P. farcta *fruit extracts, AgNPs and Ascorbic acid

**Samples**	**Concentration in mg/mL**	**Scavenging ability in %**	**IC** **50 ** **value in mg/mL**
	0.2	20.29±0.79	
	0.4	21.89±0.64	
Aqueous plant extract	0.6	25.59±1.95	1.64±0.09
	0.8	31.74±1.81	
	1	41.11±2.03	
	0.2	43.39±2.16	
	0.4	46.99±2.13	
Synthesized AgNPs	0.6	52.69±3.84	0.70±0.08
	0.8	55.89±2.26	
	1	63.56±2.41	
	0.2	50.79±1.08	
	0.4	61.89±0.60	
Ascorbic acid (Standard)	0.6	64.29±0.30	0.26±0.09
	0.8	66.99±0.30	
	1	74.49±1.20	

[Data are presented as mean ± SD (*P ***< **0.05). IC50: Concentration of the aqueous extract and synthesized nanoparticles causing 50% DPPH radical scavenging ability].

**Table 3:** Antibacterial activity of synthesized AgNPs and fruit extract of *P. farcta *against the human pathogenic bacterial strains

**Concentration(mg/mL)**					
**Culture**	**Standard**	**Fruit extract**	**AgNPs**	**AgNPs**	**Fruit extract**
		**1mg**	**1mg**	**5mg**	**5mg**
*S. aureus *(BritolA9596)	14.66±2.51	-	12±1	14±2. 5	-
*S. pneumonia *(NCTC7465)	18.66±2.08	-	11±1	13.33±0.58	-
*E. coli *(ATCC25922)	28±2.64	-	13.33±3.51	15±2.65	-
*S. typhi *(PTCC1609)	29.33±3.78	7.66±0.57	15±1.73	17.33±0.58	8.5±0.5

**Figure 1:**
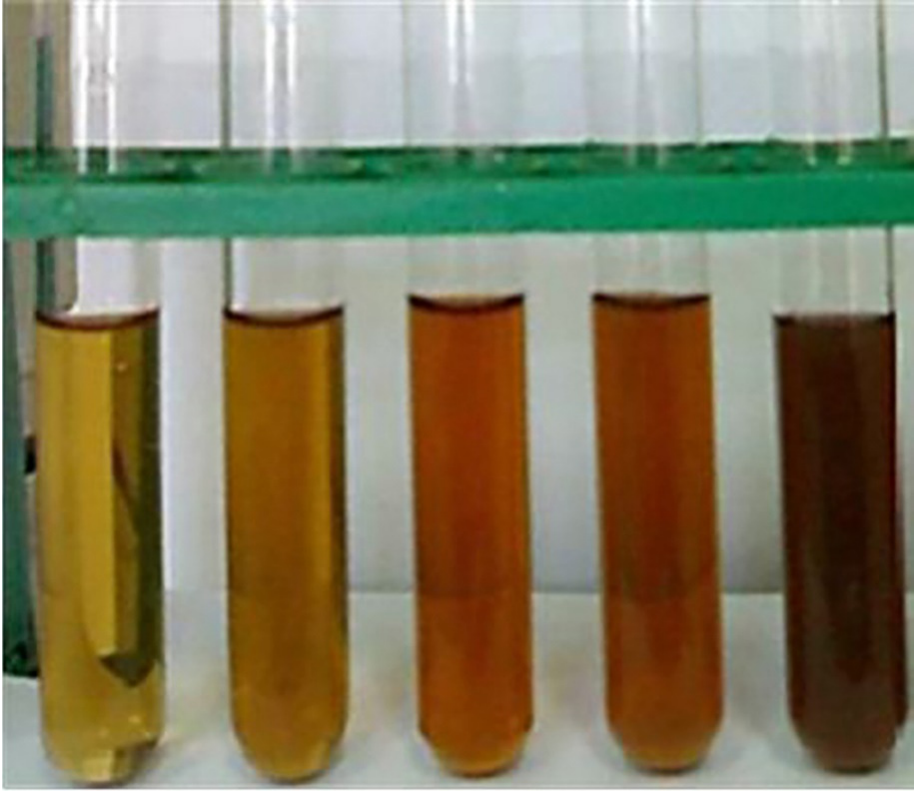
Photograph of the reduction of Ag+ to Ag in the presence of fruit extract of *P. farcta *during 12 h

**Figure 2A:**
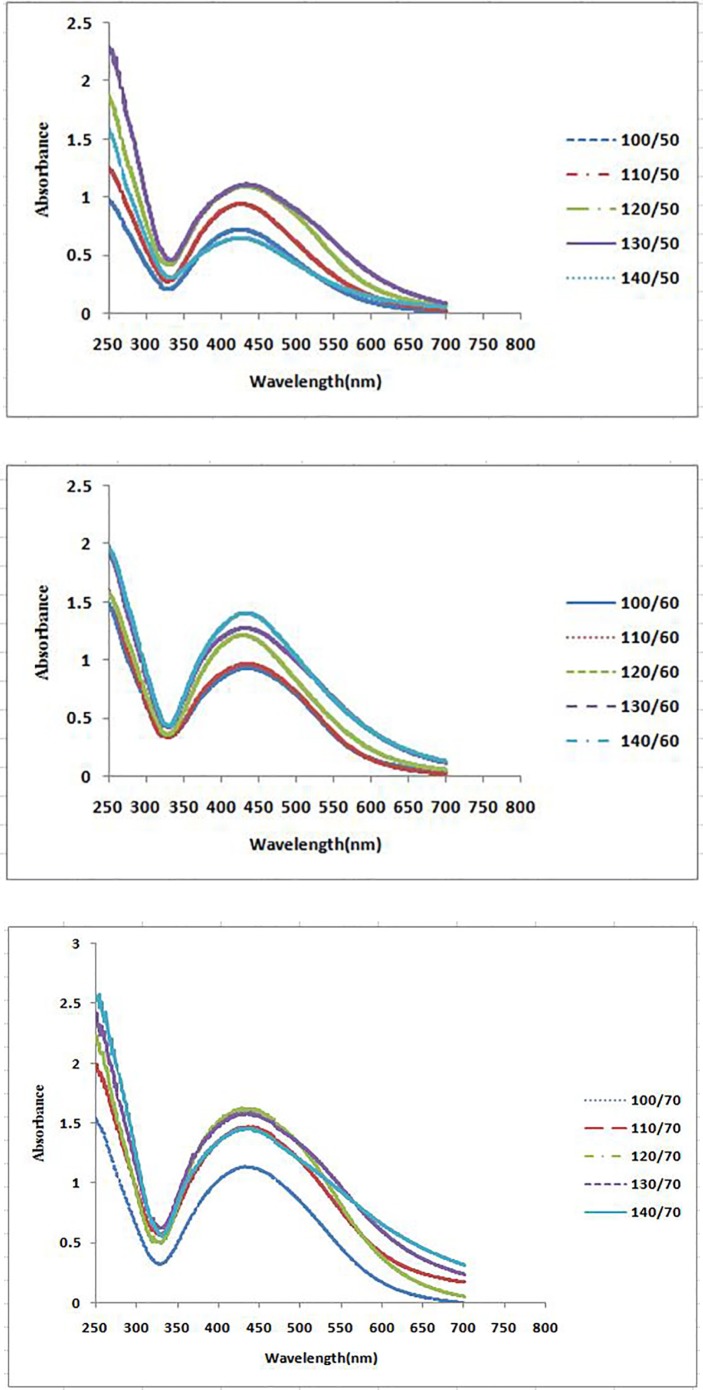
UV–vis spectra of different volumes of *P. farcta *fruit extract with aqueous solution of 0.001M AgNO3 at three different temperatures at 25 min

**Figure 2B:**
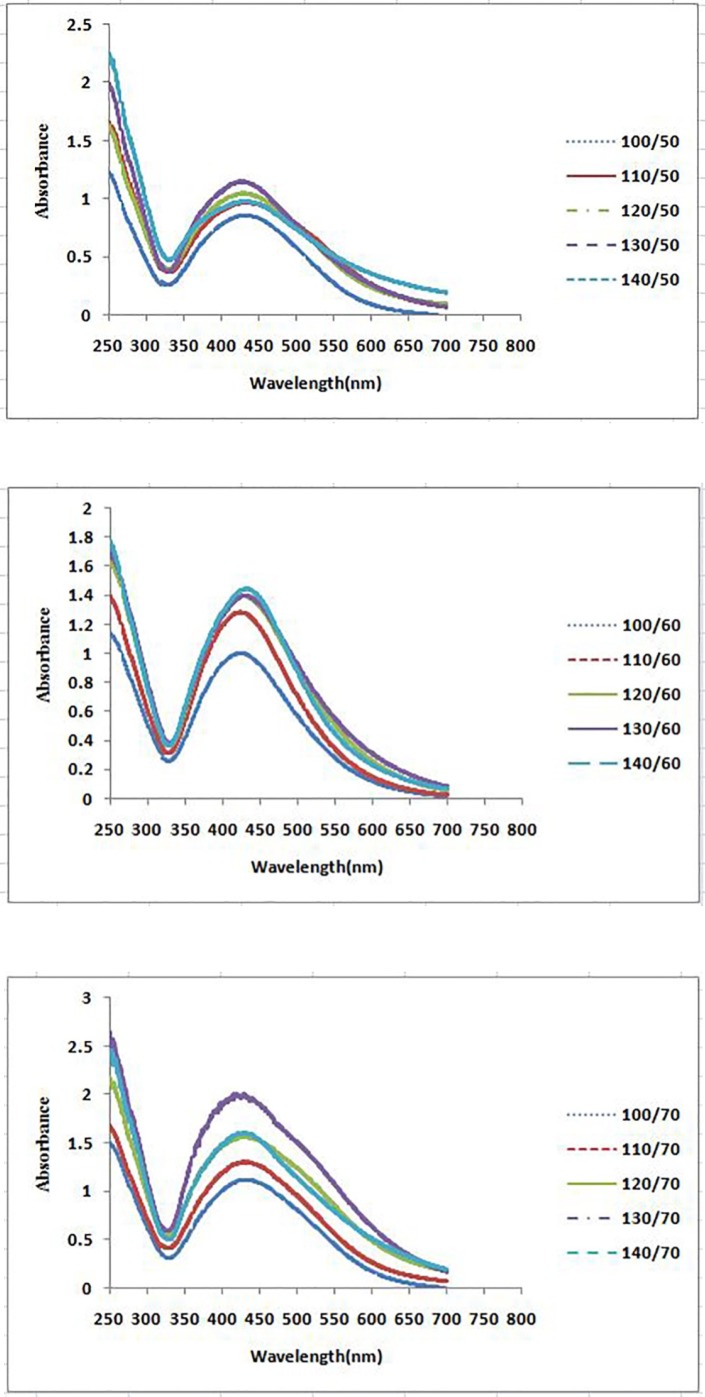
UV–vis spectra of different volumes of *P. farcta *fruit extract with aqueous solution of 0.001M AgNO3 at three different temperatures at 45 min

**Figure 3A:**
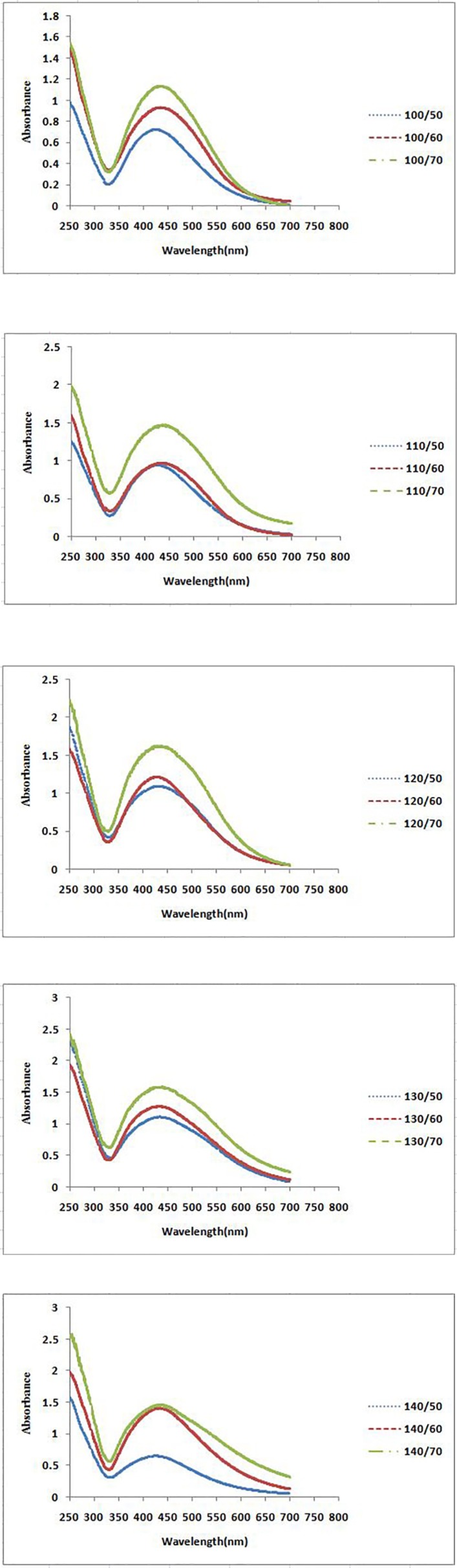
UV–vis spectra of different reaction times of *P. farcta *fruit extract with aqueous solution of 0.001M AgNO3 at three different temperatures and five different extract volumes at 25 min

**Figure 3B:**
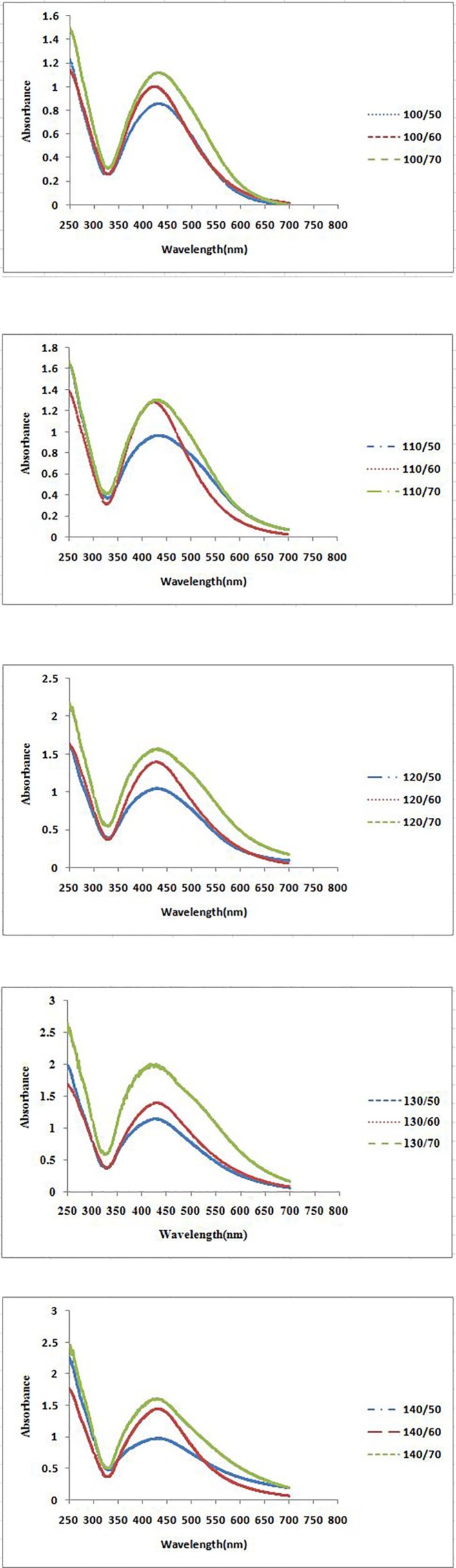
UV–vis spectra of different reaction times of *P. farcta* fruit extract with aqueous solution of 0.001M AgNO_3_ at three different temperatures and five different extract volumes at 45 min

**Figure 4:**
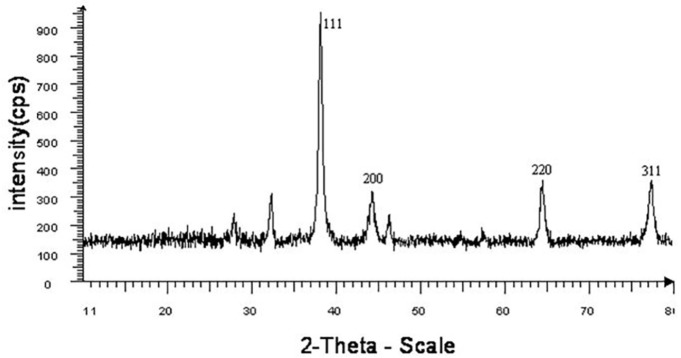
XRD spectra of synthesized MgO NPs using chemical method-I

**Figure 5:**
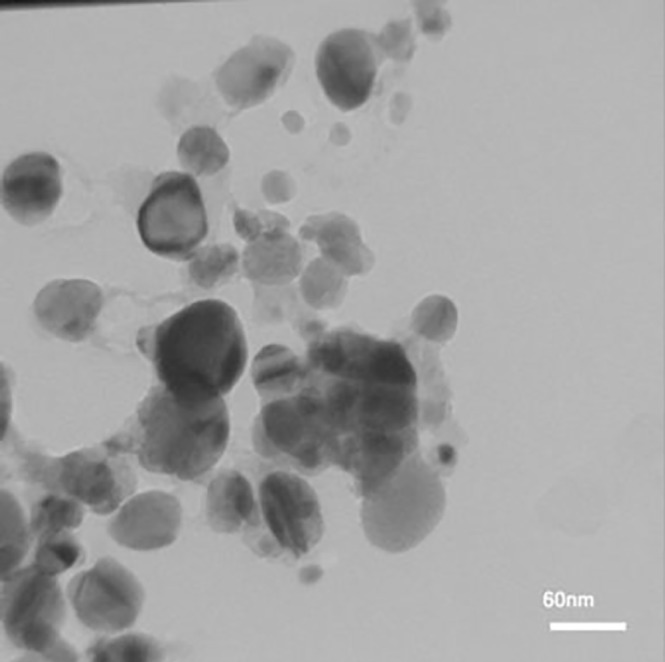
TEM image of biosynthesized silver nanoparticles

**Figure 6:**
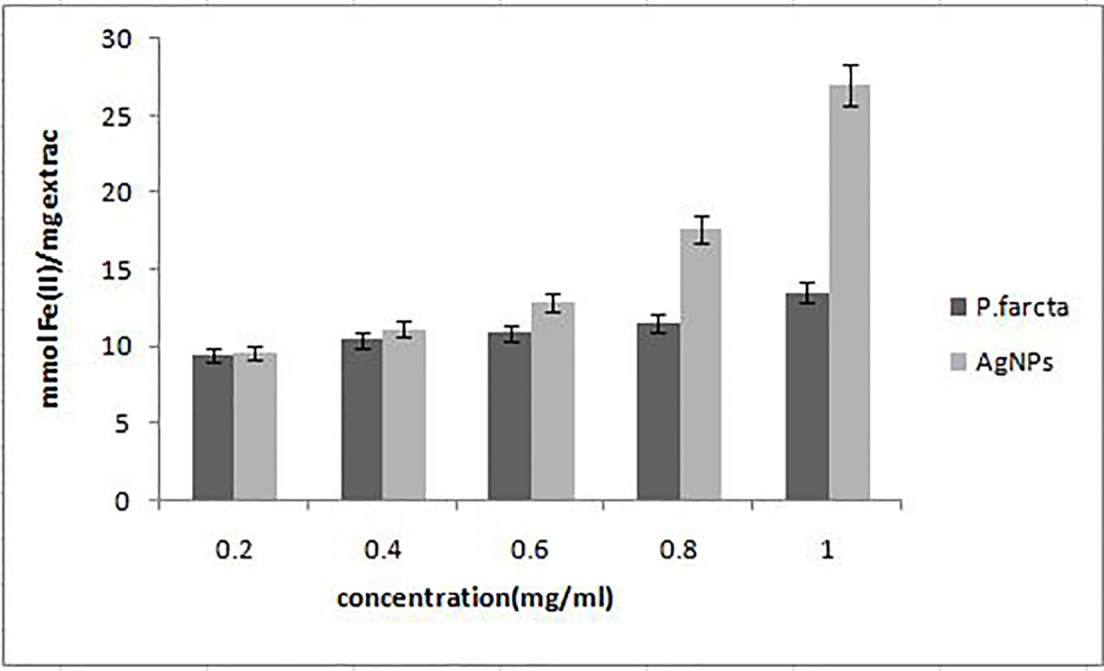
FRAP antioxidant activity of synthesized AgNPs and crude fruit extract of *P. farcta*. Gentamicin (10μg/mL) was used as a positive control. Each value represents the mean ± standard error of three replicates per treatment

**Figure 7:**
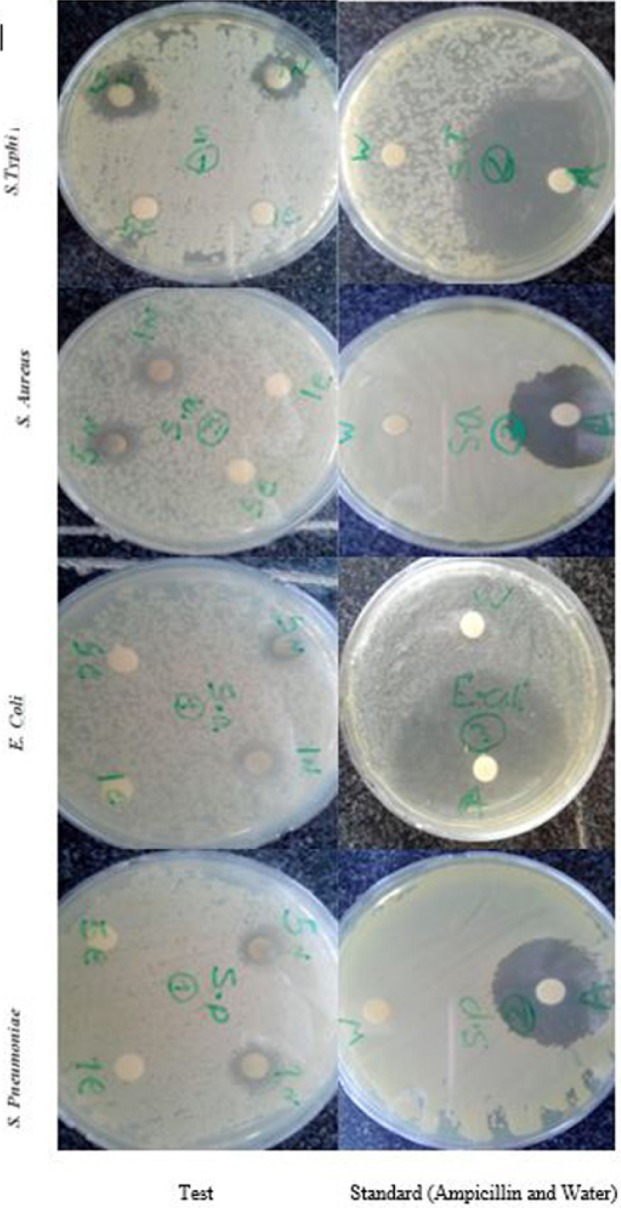
Antibacterial activity of biosynthesized Ag-NPs against human pathogens. Antibacterial effects of biosynthesized Ag-NPs evaluated by the disk diffusion method in petri plates

*TEM analysis*

Transmission electron microscopy (TEM) (Jeol JSM 2010) was recorded on a JEM-2100 with an accelerating voltage of 200 kV equipped with a high resolution CCD Camera. To prepare the samples for TEM analysis, a grid was first covered by a gold film and a small amount of the sample was dispersed in 20 mL of ethanol by sonication for 20 min, and then the grid was treated with the solution.

*Determination of total phenol content*

Total phenolic content was measured with the Folin–Ciocalteu (21). 0.1 mL of extract at appropriate dilutions was added to a mixture of 0.5 mL of Folin–Ciocalteu reagent and 0.4 mL of 7% Na2CO3 solution. The absorbance was measured at 765 nm, after incubation for 30 min in dark. The results were expressed as mg Gallic acid equivalents per gram of dry weight (mg GAE g^−1^ extract dry weight) through the standard curve with Gallic acid.

Total flavonoid content was measured according to the aluminum chloride colorimetric method as described by Chang *et al*., 2002 (22). 500 µL of aquous extract, 1.5 µL of methanol (80 %), 10% aluminum chloride (0.1 mL), 1 M potassium acetate (0.1 mL) and distilled water (2.8 mL) were mixed and incubated at room temperature for 40 min. Then, the absorbance was measured at 415 nm using spectrophotometer. Quercetin was used as a standard. The content of total flavonoids was expressed based on the mg quercetin per gram of extract dry weight.

*Determination of antioxidant activity*

*DPPH radical scavenging activity*

The biosynthesized AgNPs and the aqueous fruit extract were tested for their antioxidant activity by DPPH method (23). Each extract (0.2, 0.4, 0.6, 0.8 or 1 mg/mL) was mixed with 3 mL of methanolic solution containing DPPH radicals (0.1 mM). After 30 min, absorbance was determined at 517 nm. The percent inhibition of activity was calculated as [(Ao _ Ae)/Ao] * 100 (Ao = absorbance without extract; Ae = absorbance with extract). The results were expressed as IC50 which is the concentration of the sample required to inhibit 50 % of DPPH concentration.

*Ferric reducing antioxidant power (FRAP) assay*

FRAP assay was performed as previously described (24). The method is based on the ability of antioxidants to reduce Fe^+3^ to Fe^+2^ in the presence of TPTZ (2, 4, 6-tripyridyl-s-triazine), forming an intense blue Fe^+2^ – TPTZ complex. FRAP solution (3 mL) mixed with 100 μLof the plant extract and incubated at 37 °C for 10 min. te Absorbance was measured at 593 nm for different concentrations (0.2, 0.4, 0.8 or 1 mg/mL) of AgNPs and extract in FRAP reagent. The absorbance of the samples was compared to a FeSO_4_ standard curve and the FRAP values were expressed as mmol Fe (II)/mg extract.

*Antibacterial activity*

Antibacterial activity of the prepared fruit extract and AgNPs was evaluated using disk diffusion method (25) against common Gram positive bacteria, i.e., *Staphylococcus aureus* (Britol A9596), *Streptococcus pneumoniae* (NCTC 7465) and Gram-negative bacteria, i.e., *Escherichia coli* (ATCC 25922), *Salmonella typhi *(PTCC 1609). The pure bacterial cultures were cultured on nutrient broth. The plates were left overnight at room temperature to allow any contamination to appear. Sterile paper disks were placed on Muller Hinton agar plates inoculated with each of the mentioned microorganisms.1 mg/mL and 5 mg/mL of Ag-NPs and fruit extracts were placed on inoculated agars. The test plates were incubated at 37 °C for 24 h. The zones of inhibition were measured and numbers reported in average (Table 3). Gentamicin was used as an antibacterial standard against all pathogens.

## Results and Discussion

Reduction of Ag^+^ into silver nanoparticles during exposure to the *P. farcta* fruit extract could be observed by the color change. The color of fresh prepared fruit extract of *P. farcta *was yellow. After the addition of AgNO_3_ to the extract and incubation for 6 h in a rotary shaker at 150 rpm, the extract color was turned dark brown (Figure 1). Color changes in aqueous solutions are due to the surface plasmon resonance phenomenon (25). The results were shown that the fruit extract of *P. farcta* is a good potential reducing agent for Ag^+^ ions. In accordance with the results of Shameli *et al*. (26), plausible chemical equations for the biosynthesis of silver nanoparticles are followings:

Ag^+ ^_(aq) _+ *P. farcta*_(aq)_→[Ag (*P. farcta*)]^+ ^                            Equ.1 

[Ag (*P. farcta*)] ^+^ + R-CHO→[Ag (*P. farcta*)] + R-COOH                            Equ.2 

After dispersion of silver ions in the aqueous fruit extract of *P. farcta* (Equ. 1), the complex of [Ag (*P. farcta*)] ^+^ was reacted with aldehyde groups present in the natural products structure to obtain [Ag (*P. farcta*)] due to the reduction of silver ions via oxidation of aldehyde to carboxylic acid groups (Equ. 2).

*Optimization study*

*Influence of extract concentration*

The formation process of AgNPs was detected and followed by measuring the surface plasmon resonance (SPR) of *P. farcta* extract and Ag/* P. farcta* suspensions over the wavelength range of 250 to 700 nm at 50, 60 and 70 °C at 25 and 45 min (Figures 2, 3). SPR bands are affected by the composition, morphology, shape, size and dielectric environment of prepared nanoparticles (27, 28). It was proven that spherical silver nanoparticles contribute to the absorption bands around 425–475 nm in the UV–visible spectra (29). UV–visible spectra of nanoparticles were obtained on varying the *P. farcta* fruit extract volumes at 25 min reaction time. On increasing the volume from 100 µL to 130 µL at 50 °C, λ_max_ was increased from 425 to 438 nm with an increase in the absorbance from 0.721 to 1.111. On further increasing the concentration to 140 µL, a decreasing in λ_max_ to 425 nm with decreasing in the absorbance to 0.647 was observed. Therefore, for further experiments, the volume of 130 µL of *P. farcta* fruit extract at 25 min was selected. The size of silver nanoparticles depends on the ratio of Ag^+^ and reducing stabilizing agent. The slight variations in λ_max_ values signify changes in particle size owing to the change in concentration ratios between *P. farcta* fruit extract and Ag^+^. Similar evaluation was accomplished at 45 min reaction time. On increasing the volume from 100 µL to 130µL at 50 °C, λ_max_ was increased from 430 to 438 nm with an increase in the absorbance from 0.857 to 1.146. On further increasing the concentration to 140 µL, a small change in λ_max_ to 431 nm was observed with decreasing the absorbance to 0.978.

*XRD analysis*

XRD as a powerful nondestructive technique for characterizing crystalline materials provides information on structures, phases, preferred crystal orientations, and other structural parameters, such as average grain size, crystallinity, strain, and crystal defects. XRD pattern clearly exhibited the presence of silver nanoparticles (Figure 4). XRD pattern showed four distinct diffraction peaks at 38, 44, 64.3, and 77.1 which were pertained to (111), (200), (220), and (311) of AgNPs, respectively. All these diffraction peaks can be perfectly indexed to the face-centered cubic (FCC) crystalline structure of Ag, not only in peak position, but also in their relative intensity of the characteristic peaks. The average crystallite size of silver nanoparticles was obtained using Scherrer’s Equation:

In which, D is the crystallite size, k is the shape factor that assumes a value of 0.89 for Ag, λ is the X-ray wavelength (1.5406 A˚), β is the half height width of XRD peak and θ is the diffraction angle. The diameters of silver nanoparticles were estimated to be at the range 10.26-14.65 nm for all samples.

*TEM analysis*

To investigate the morphology and the particle size of AgNPs, TEM image was taken (Figure 5). A good correlation between the particle size obtained from Scherrer equation and TEM image was observed.

*Total phenolic and flavonoid content*

The results of total phenolic content (TPC) of fruit extract alone or plus AgNPs showed that TPC was higher in plant- AgNPs (462.69 ± 3.42 mg/g GAE) compared to the aqueous fruit extract alone (366.21 ± 3.03 mg/g GAE) (Table 1). The results also revealed that total flavonoids were higher in plant-AgNPs compared to those found in the fruit extract alone (Table 1). Compounds such as phenolics, flavanoids, terpenoids, and soluble proteins have been reported to act as capping agents (30). Similar to our results, Abdel-Aziz *et al*., (2014) and Sultana *et al*., (2015) (31, 32) reported a higher total phenol and flavonoid content in synthesized AgNPs compared to the *Chenopodium murale *and *Houttuynia cordata* leaf extract respectively.

The antioxidant activity of synthesized AgNPs and aqueous fruit extract was determined by using DPPH free radical and FRAP assay. DPPH is a stable compound which can be reduced by accepting the hydrogen or electrons and has been widely used to evaluate the antioxidant activity (33). The lower IC50 value indicates a stronger ability of the extract to act as a DPPH scavenger, while the higher IC50 value indicates a lower scavenging activity. The effect of different concentrations of AgNPs on DPPH radical antioxidant activity is shown in Table 2. Our results revealed that the aqueous fruit extract and synthesized AgNPs are free radical scavengers. However, the AgNPs exhibited more scavenging activity of DPPH than aqueous fruit extract. The DPPH activity of the AgNPs and fruit extract was found to increase in adose-dependent manner. At concentrations 0.2–1 mg/mL, AgNPs showed a scavenging activity ranging from 43% to 63% with average IC50 value, 0.70 ± 0.08. The antioxidant activity was lower than that of standard ascorbic acid at 1 mg/mL (74%). In FRAP assay, the ability of AgNPs to reduce Fe^3+^ to Fe^2+^ was also significantly higher than that of *P. faracta* extract at concentrations 0.2–1 mg/mL (Figure 6).

There are few reports on the antioxidant activity of the biosynthesized AgNPs. AgNPs synthesis, characterization and antioxidant activities, was reported in* Fraxinus excelsior* leaf extract (34), *Terminalia* species leaf extract (35), *Elephantopuss caber* (36), *Cleistanthus collinus* [37]. Our result showed that *P. faracta* is a good source of phenolic compounds and flavonoids. Phenolic and flavonoids have been reported to be the most important phytochemicals responsible for the antioxidant capacity (38). In this research the nanoparticles synthesized using fruit extract of *P. faracta* showed antioxidant activity due to capped phenolic compounds. Phenolic group facilitates the conversion of silver nitrate to AgNps due to its electron donating ability (39).

*Antimicrobial activity*

The antibacterial effect of biosynthesized AgNPs (1 & 5 mg/mL) was investigated against various pathogenic organisms such as *S. typhi*, *E. coli, S. aureus *and *S. pneumoniae*. AgNPs showed the significant antibacterial activity on all the four bacterial strains tested when compared to the fruit extract (Figure.7). Moreover, AgNPs exhibit effective zone of inhibition against gram-negative bacteria (*S. typhi, E. coli*) compared to the gram-positive bacteria (*S. aureus *and *S. pneumoniae*). Among the various tested bacterial the highest zone of inhibition (17.33 ± 0.58 mm) was recorded by *S. typhi* and least zone of inhibition (11 ± 1 mm) was recorded with *S. pneumoniae* (Table 3). Saravanakumar *et al*., (2015) also reported* Cassia tora* AgNPs had higher bacterial activity against gram-negative bacteria compared to the gram-positive bacteria (40). The potential reason for the antibacterial activity of silver is that AgNPs may attach to the surface of the cell membrane disturbing permeability and respiration functions of the cell. It is also possible that AgNPs not only interact with the surface of the membrane, but can also penetrate inside the bacteria. The higher AgNPs antibacterial activity against gram negative bacteria is due to their thinner peptidoglycan layer which can easily enter in to cell wall to denature or kill bacteria (41).

## Conclusion

The current study revealed that silver nanoparticles can be synthesized in a simple method using* P. farcta* fruit extract. The TEM analysis showed that the sizes of the synthesized AgNps ranged from 10.26 to14.65 nm. The total phenolic compounds and total flavonoids were higher in plant-AgNPs compared to the plant extract alone. Plant-AgNPs showed a higher antioxidant and antibacterial activity compared to *P. farcta* fruit extract alone.

## References

[1] Tolaymat TM, Badawy AME, Genaidy A, Scheckel KG, Luxton TP, Suidan M (2010). An evidence-based environmental perspective of manufactured silver nanoparticle in syntheses and applications: a systematic review and critical appraisal of peer-reviewed scientific papers. Sci. Total Environ.

[2] Rai M, Yadav A, Gade A (2009). Silver nanoparticles as a new generation of antimicrobials. Biotechnol. Adv.

[3] Sathishkumar G, PK Jha, Vignesh V, Rajkuberan C, Jeyaraj M, Selvakumar M, Jha R, Sivaramakrishnan S (2016). Cannonball fruit (Couroupita guianensis) extract mediated synthesis of gold nanoparticles and evaluation of its antioxidant activity. J. Mol. Liquids.

[4] Nanda BA, Saravanan M (2009). Biosynthesis of silver nanoparticlesfrom Staphylococcus aureus and its antimi crobial activityagainst MRSA and MRSE. Nanomedicine.

[5] Njagi EC, Huang H, Stafford L, Genuino H, Galindo HM, Collins JB, Hoag GE, Suib S (2011). Biosynthesis of iron and silver nanoparticles at room temperature using aqueous sorghum bran extracts. Langmuir.

[6] Muthukrishnan S, Bhakya S, Senthil Kumar T, Rao MV (2015). Biosynthesis, characterization and antibacterial effect of plant-mediated silver nanoparticles using Ceropegia thwaitesii–An endemic species. Ind. Crops. Prod.

[7] Mervat F, Zayed WH, Eisa WH, Shabaka AA (2012). Malva parviflora extract assisted green synthesis of silver nanoparticles. Spectrochim. Acta Mol. Biomol. Spectros.

[8] Kuppusamy P, Yusoff MM, Govindan N (2015). Biosynthesis of metallic nanoparticles using plant derivatives and their new avenues in pharmacological applications – An updated report. Saudi Pharm. J.

[9] Mahendran G, RanjithaKumari DR (2016). Biological activities of silver nanoparticles from Nothapodytesnim moniana (Graham) Mabb Fruit extracts. Food Sci. Human Wellness.

[10] Moghaddam MG, Dabanlou RH (2014). Dabanlou, Plant mediated green synthesis and antibacterialactivity of silver nanoparticles using Crataegus douglasii fruit extract. J. Ind. Eng. Chem.

[11] Subashini R, Sruthi S, Sindhuja P, Santhini S, Gnana Prakash D (2015). Biosynthesis of Silver Nanoparticles using Garcinia mangostana fruit extract and their antibacterial, antioxidzant activity. Int.J. Curr. Microbiol. App. Sci.

[12] Medhi M (2014). Eco-friendly synthesis of silver nanoparticles using fruit extract of Averrhoa Carambola. Int. J. Innov. Sci. Eng. Technol.

[13] Kumar B, Smita K, Cumbal L, Debut A (2015). Lantana camara berry for the synthesis of silver nanoparticles. Asian Pac. J. Trop. Biomed.

[14] Ramesh PS, Kokila T, Geetha D (2015). Plant mediated green synthesis and antibacterial activity of silver nanoparticles using Emblica officinalis fruit extract, Spectrochim Spectrochim. Acta Mol. Biomol. Spectrosc.

[15] Pasiecznik NM, PJC Harris, Smith SJ (2004). Identifying Tropical Prosopis Species: A Field Guide.

[16] Nahal-Tahmasbi MR (2000). Ecological study of American (Pukestanian) Kahoor and effectiveness for compost production in Hormozgan province. Agric. Econ. Develop.

[17] Harzallah-Skhiri F, BenJannet H (2005). Flavonoids diversification in organs of two Prosopis farcta (banks& sol) eig (leguminosea mimosoideae) populations occurring in the northeast and the southeast of Tunisia. J. Appl. Sci. Res.

[18] Sharma N, Garg V, Paul A (2010). Antihyperglycemic, antihyperlipidemic and antioxidative potential of Prosopis cineraria bark. Indian.J. Clinical. Biochem.

[19] Rahman AA, Samoylenko V, Jacob MR, Sahu R, Jain SK, Khan SI, Tekwani BL, Muhammad I (2011). Antiparasitic and antimicrobial indolizidines from the leaves of Prosopis glandulosa var. glandulosa. Planta Med.

[20] Robertson S, Narayanan N, Raj Kapoor B (2011). Antitumour activity of Prosopis cineraria (l) druce against ehrlich ascites carcinoma-induced mice. Nat. Prod. Res.

[21] McDonald S, Prenzler PD, Antolovich M, Robards K (2001). Phenolic content and antioxidant activity of olive extracts. Food Chem.

[22] Chang CC, Yang MH, Wen HM, Chern JC (2002). Estimation of total flavonoid content in propolis by two complementary colorimetric methods. JFDA.

[23] Shimada K, Fujikawa K, Yahara K, Nakamura T (1992). Antioxidative properties of xanthin on auto oxidation of soybean oil in cyclodextrin emulsion. Agri. Food Chem.

[24] Guo C, Yang J, Wei J, Li Y, Xu J, Jiang Y (2003). Antioxidant activities of peel, pulp and seed fractions of common fruits as determined by FRAP assay. Nut. Res.

[25] Raja Naika H, Lingaraju K, Manjunath K, Danith Kumar, Nagaraju G, Suresh D, Nagabhushana H (2015). Nagabhushana, Green synthesis of CuO nanoparticles using Gloriosa superba L extract and their antibacterial activity. J. Taibah Univ. Sci.

[26] Shameli K, Bin Ahmad M, Jaffar E A, Mulla A, Ibrahim NA, Shabanzadeh P, Rustaiyan A, Abdollahi Y, Bagheri S, Abdolmohammadi S, Sani Usman M, Zidan M (2012). Green Biosynthesis of Silver Nanoparticles Using Callicarpa maingayi Stem Bark Extraction, Molecules. Molecules.

[27] Kelly KL, Coronado E, Zhao LL, Schatz GC (2003). The Influence of Size, Shape, and Dielectric Environment. J. Phys. Chem. B.

[28] Stepanov AL (1997). Optical properties of metal nanoparticles synthesized in a polymer by ion implantation. Tech. Phys.

[29] Stamplecoskie KG, Scaiano JC (2010). Light Emitting Diode Irradiation Can Control the Morphology and Optical Properties of Silver Nanoparticles. J. Am. Chem. Soc.

[30] Ramamurthy Ch, Padma M, Mareeswaran R, Suyavaran A, Kumar MS, Premkumar K, Thirunavukkarasu C (2013). The extra cellular synthesis of gold and silver nanoparticles and their free radical scavenging and antibacterial properties. Colloids Surf. B: Biointerfaces.

[31] Abdel-Aziz MS, Shaheen MS, El-Nekeety AA, Abdel-Wahhab MA (2014). Abdel-Wahhab, Antioxidant and antibacterial activity of silvernanoparticles biosynthesized using Chenopodium murale leaf extract. J. Saudi Chem. Soc.

[32] Sultana F, Barman J, MousmiSaikia B (2015). biological approach to synthesis of silver nanoparticles using aqueous leaf extract of Houttuynia cordata Thunb and comparative antioxidant study of plant extract and synthesized nanoparticles. J. Mater. Bio. Appl.

[33] Mittal AK, Bhaumik J, Kumar S, Banerjee UC (2014). Biosynthesis of silver nanoparticles: Elucidation of prospective mechanism and therapeutic potential. J. Colloid Interface Sci.

[34] Parveen M, Ahmad F, Malla AM, Azaz S (2016). Microwave-assisted green synthesis of silver nanoparticlesfrom Fraxinus excelsior leaf extract and its antioxidant assay, Microwave-assisted green synthesis of silver nanoparticles from Fraxinus excelsior leaf extract and its antioxidant assay. Appl. Nanosci.

[35] El-Rafie HM, Hamed M (2014). Antioxidant and anti-inflammatory activities of silver nanoparticles biosynthesized from aqueous leaves extracts of four Terminalia species. Adv. Nat. Sci: Nanoscie. Nanotechnol.

[36] Kharat SN, Mendhulkar VD (2016). Mendhulkar. Synthesis Characterization and studies on Antioxidant activity of Silver Nanoparticles using Elephantopuss caber leaf extract. Mater. Sci. Eng.

[37] Kanipandian N, Kannan S, Ramesh R, Subramanian P, Thirumurugan R (2014). Antioxidant and cytotoxicity evaluation of green synthesized silver nanoparticles using Cleistanthus collinus extractas surface modifier. Mater. Res. Bulletin.

[38] Saumya S, Basha P (2011). Antioxidant effect of Lagerstroemia speciosa Pers (Banaba) leaf extract in streptozotocin-induced diabetic mice. Indian J. Exp. Biol.

[39] Philip D (2011). Mangifera indica leaf-assisted biosynthesis of well-dispersed silver nanoparticles Spectrochim. Acta Part A: Mol. Biomol. Spectroc.

[40] Saravanakumar A, Ganesh M, Jayaprakash J, Jang HT (2015). Biosynthesis of silver nanoparticles using Cassia tora leaf extract andits antioxidant and antibacterial activities. J. Ind. Eng. Chem.

[41] Asmathunisha N, Kathiresan K, Anburaj, Nabeel MA (2010). Synthesis of antimicrobial silver nanoparticles by callus and leaf extracts from saltmarsh plant, Sesuvium portulacastrum L. Coll. Surf. B: Biointer.

